# Protective Role of Acidic pH-Activated Chloride Channel in Severe Acidosis-Induced Contraction from the Aorta of Spontaneously Hypertensive Rats

**DOI:** 10.1371/journal.pone.0061018

**Published:** 2013-04-08

**Authors:** Zhiyong Ma, Jia Qi, Zhijie Fu, Mingying Ling, Li Li, Yun Zhang

**Affiliations:** 1 Key Laboratory of Cardiovascular Remodeling and Function Research, Chinese Ministry of Education and Chinese Ministry of Public Health; Department of Cardiology, Qilu Hospital, Shandong University, Jinan, China; 2 Department of Otorhinolaryngology, Shandong Provincial Qianfoshan Hospital, Clinical Medical College of Shandong University, Jinan, China; The University of Manchester, United Kingdom

## Abstract

Severe acidic pH-activated chloride channel (I_Cl,acid_) has been found in various mammalian cells. In the present study, we investigate whether this channel participates in reactions of the thoracic aorta to severe acidosis and whether it plays a role in hypertension. We measured isometric contraction in thoracic aorta rings from spontaneously hypertensive rats (SHRs) and normotensive Wistar rats. Severe acidosis induced contractions of both endothelium-intact and -denuded thoracic aorta rings. In Wistar rats, contractions did not differ at pH 6.4, 5.4 and 4.4. However, in SHRs, contractions were higher at pH 5.4 or 4.4 than pH 6.4, with no difference between contractions at pH 5.4 and 4.4. Nifedipine, I_Cl,acid_ blockers 5-nitro-2-(3-phenylpropylamino) benzoic acid (NPPB) and 4,4′-diisothiocyanatostilbene-2, 2′-disulfonic acid (DIDS) inhibited severe acidosis-induced contraction of aortas at different pH levels. When blocking I_Cl,acid_, the remnant contraction was greater at pH 4.4 than pH 5.4 and 6.4 for both SHRs and Wistar rats. With nifedipine, the remnant contraction was greatly reduced at pH 4.4 as compared with at pH 6.4 and 5.4. With NPPB or DIDS, the ratio of remnant contractions at pH 4.4 and 5.4 (R_4.4/5.4_) was lower for SHRs than Wistar rats (all <1). However, with nifedipine, the R_4.4/5.4_ was higher for SHRs than Wistar rats (both >1). Furthermore, patch clamp recordings of I_Cl,acid_ and intracellular Ca^2+^ measurements in smooth muscle cells confirmed these findings. I_Cl,acid_ may protect arteries against excess vasoconstriction under extremely acidic extracellular conditions. This protective effect may be decreased in hypertension.

## Introduction

Extracellular pH (pH_o_) is generally maintained within a narrow range between 7.35 and 7.45, but some pathological conditions, such as ischemia, hypoxia, metabolic disorders, gastrointestinal disorders and renal dysfunction may cause local or systemic extracellular acidification [1], [2]. Increasing evidence reveals that extracellular acidosis could modulate vascular tone and play an important role in hypertension [3]–[5]. Furukawa *et al.* [3] found that slightly acidic pH induced contraction of aortas from both spontaneously hypertensive rats (SHRs) and Wistar Kyoto rats. However, acidosis induced relaxation mediated by nitric oxide and potassium channels in rat thoracic aortas pre-contracted with phenylephrine [4]. The different results may be induced by different levels of acidosis and different exposure times used in these studies.

Recently, a novel type of chloride channel activated by severe acidic solution was found in various mammalian cell types such as HEK293 cells [6], cardiac myocytes [7], and monocytes [8]. This channel was activated by very acidic extracellular conditions (pH <5.5) and exhibited an outward rectification in the I–V relationship and activation independent of intracellular Ca^2+^ [6]–[8]. Our previous study also found this channel in human umbilical vein endothelial cells [9]. However, whether this channel plays an important role in the reactions of the rat thoracic aorta to severe acidosis and in hypertension is unclear.

In the present study, we compared the isometric contractions of thoracic aorta rings from SHRs and normotensive Wistar rats in different pH solutions (pH 7.4, 6.4, 5.4 and 4.4) to reveal the different reactions of the rat thoracic aorta to severe and extreme acidosis and to explore whether these reactions are changed in hypertension. We studied the role of I_Cl,acid_ in severe acidosis-induced aortic contraction and hypertension.

## Methods

### Experimental Animals

Male SHR and age-matched Wistar rats (12 to 13 weeks old) were used (n = 6 for each group). Animals were housed in an animal holding facility under standard light (12-h light/dark cycle), temperature (22±0.5°C), and humidity (60±10%). Before rats were killed, systolic blood pressure (SBP) was measured by the tail-cuff method (MK-2000, Muromachi, Tokyo, Japan). All animal care and procedures were approved by the Animal Care Committee of Shandong University and complied with the Guide for the Care and Use of Laboratory Animals by the US National Institutes of Health.

### Measurement of Isometric Tension

Rats were anesthetized by intraperitoneal pentobarbital injection, and descending thoracic aortas were removed. The aortas were cleared of connective tissue and cut into rings (2–3 mm in length) in oxygenated physiological salt solution (PSS; in mM: 130 NaCl, 5 KCl, 1.2 MgCl_2_, 1.5 CaCl_2_, 10 HEPES and 8 glucose), which was titrated to pH 7.4 with NaOH and constantly oxygenated with 100% O_2_.

The aorta rings were placed between 2 stainless steel wires in a 5-ml organ bath (DMT 610 M, Danish Myo Technology, Denmark) filled with PSS, which was maintained at 37°C and bubbled with 100% O_2_. The isometric force of aorta rings was recorded by use of a Powerlab system (ML-845, AD Instruments, Australia). Each ring was stretched to the optimal length–tension of 2.0 g and allowed to equilibrate for 30 min.

The endothelium of aorta rings was removed by gently rubbing the endothelial surface with cotton pellets. It was considered present when the acetylcholine (10 µM)-induced relaxation was at least 80% after pre-contraction with 30 mM KCl salt solution and absent with no relaxation response. The aorta rings were pre-contracted 3 times with 30 mM KCl salt solution, and the last plateau the contraction was considered a reference. Then each ring was washed and re-equilibrated for 30 min.

### pH–response Curves for Rat Aorta Rings

For pH-dependence analysis, aorta rings were incubated in bath solutions of pH 7.4, 6.4, 5.4 and 4.4 sequentially. Previous studies found that I_Cl,acid_ was activated at pH <5.5 and usually used different pH levels to study the characteristics of I_Cl,acid_ [6]–[8]. We chose the following pH range: 7.4 (normal), 6.4 (acidic pH cannot induce I_Cl,acid_), 5.4 (threshold pH induces I_Cl,acid_) and 4.4 (induces large I_Cl,acid_). pH values were changed by adding HCl (0.5 M) to the organ bath and monitored by a pH electrode connected to a pH-meter (Thermo Orion 920A+, Thermo Scientific, USA), which enabled real-time measurement of the solution pH simultaneously with tension recording. The time interval between consecutive additions of HCl was 15 min, which was necessary to observe the contractile responses to pH-changes and to let the contraction reach a stable plateau. To not affect the osmotic pressure of the bath solution, the total final volume of HCl added to the organ bath was about 11 µl.

To study the mechanism of acidosis-induced vasoconstriction, the acidic pH-response curves of the rings were also examined in the presence of chloride channel blockers: 5-nitro-2- (3-phenylpropylamino) benzoic acid (NPPB, 100 µM) and 4,4′-diisothiocyanatostilbene-2, 2′-disulfonic acid (DIDS, 100 µM). Severe acidosis-induced contraction was recorded with use of the voltage-dependent calcium channel blocker (VDCC) nifedipine (10 µM) and calcium-free solution. The aorta rings were treated with different agents 30 min before changing the pH of the bath solution from 7.4 to 6.4 or even lower. Because nifedipine is light sensitive, experiments involving it were performed in the dark.

### Calculating the Ratio between Remnant Contractions at pH 4.4 and 5.4

Because previous studies found that I_Cl,acid_ is usually activated at pH <5.5, the difference between contractions at pH 5.4 and 4.4 might reflect the effect of I_Cl,acid_ in severe acidosis-induced contraction. We normalized this difference by calculating the ratio of the remnant contractions at pH 4.4 and 5.4 (R_4.4/5.4_). R_4.4/5.4_>1 indicated that the aorta rings contracted further with decreasing pH from 5.4 to 4.4. R_4.4/5.4_<1 indicated that aorta rings relaxed.

### Smooth Muscle Cell (SMC) Isolation

Descending thoracic aortas were removed as described above and placed in free-Ca^2+^ PSS supplemented with 1 mg/ml fat-free bovine serum albumin (Sigma Chemical, St. Louis, MO). Arteries were cleaned of connective tissue and transferred to a vial containing 1 ml of the same solution with papain (1.5 mg/ml) and dithioerythritol (1 mg/ml) for 30 min at 37°C. The tissue was then incubated in 1 ml of fresh free-Ca^2+^ solution containing collagenase (type F, 1 mg/ml) for an additional 15 min. Then the arteries were placed in enzyme-free solution and triturated through a Pasteur pipette until single SMCs were observed under a microscope. SMCs were stored in PSS at 4°C until use. Cell viability was assessed by trypan blue exclusion as described in Methods S1.

### Patch Clamp Recordings

SMCs were subcultured onto glass coverslips for at least 10 min before patch clamping. Patch clamp recording was as we described previously [8], [9]. To induce I_Cl,acid_, SMCs were perfused with pH 7.4 or 4.4 solutions. To investigate the effect of different drugs on the currents, cells were perfused with bath solutions at pH 7.4, 4.4, and 4.4 plus agents.

### Intracellular Calcium Measurements by Calcium Imaging

SMCs were incubated with 2 µM fura-2/acetoxymethylester for 1 h at 37°C, then 30 min of washout at room temperature. Calcium imaging involved a dual excitation wavelength fluorescence method, with a TILLvisION digital imaging system (TILL Photonics GmbH, Munich, Germany) and a Nikon inverted microscope with a ×40 oil immersion objective, as reported previously [10], [11]. Intracellular calcium ([Ca^2+^]_i_) concentration was indicated as the ratio of fluorescence intensity at 340 and 380 nm (Ratio_(340/380)_), with an emission wavelength at 510 nm. Background fluorescence intensity was corrected. To induce I_Cl,acid_, SMCs were perfused with pH 7.4 or 4.4 solutions for 1.5 min each. To investigate the effect of severe acidosis and different drugs on [Ca^2+^]_i_, cells were perfused with bath solutions at pH 7.4, 4.4, and 4.4 plus agents. [Ca^2+^]_i_ at different pH or with drugs was normalized to that at pH 7.4.

### Reagents

Acetylcholine (10 mM) was dissolved in deionized water. Stock solutions of nifedipine, DIDS, NPPB and fura-2/acetoxymethylester were prepared in dimethylsulphoxide (DMSO) at 10 mM. All chemicals were from Sigma (St. Louis, MO, USA) and diluted on the day of the experiment in fresh solution.

### Statistical Analysis

Data are expressed as mean ±SE. Contraction in different treatments was normalized to the 30-mM KCl-induced contraction. The pH-response curves were fitted by use of the following equation: contraction = a/(1+(EC_50_/pH)^nH^), where a is the amplitude of contraction, EC_50_ is the pH at which a half-maximal response was induced, and nH is the Hill coefficient. The unpaired Student’s *t* test and one-way ANOVA with repeated measures were used for statistical analysis as appropriate. *P*<0.05 was considered statistically significant.

## Results

### Effect of Severe and Extreme Acidosis on Thoracic Aorta Contractions

**Figure 1:**
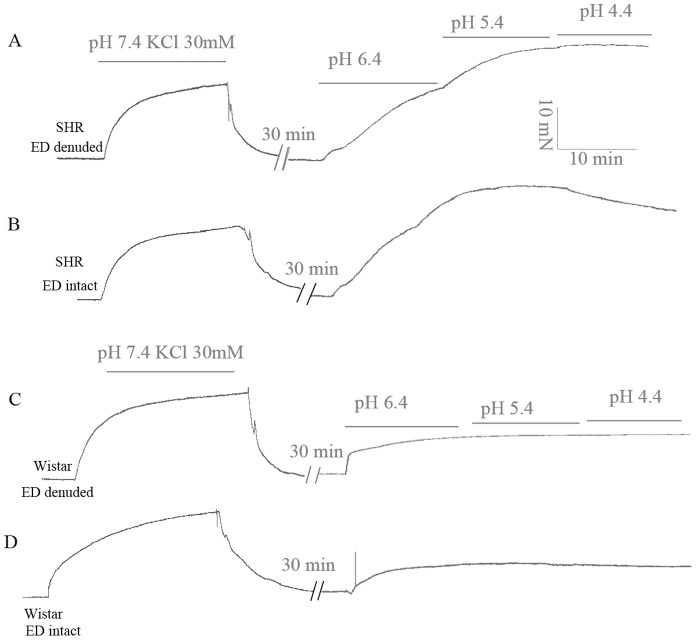
Representative recordings showing acidic pH-induced aorta contraction for spontaneously hypertensive rats (SHRs, n = 6) and Wistar rats (n = 6). Extreme and severe acidosis induced contraction of both endothelium-denuded (ED-denuded; A, C) and endothelium-intact (ED-intact; B, D) thoracic aorta rings.

**Figure 2:**
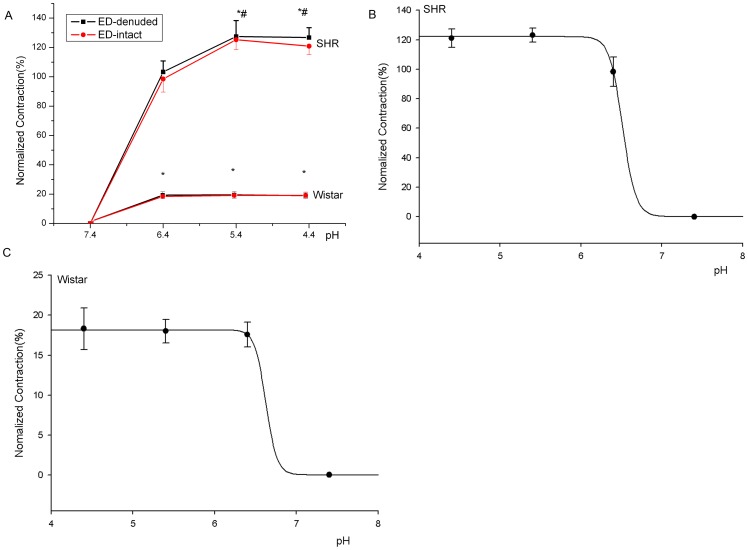
Effect of extreme and severe acidosis on thoracic aorta contraction for SHRs (n = 6) and Wistar rats (n = 6). A, Extreme and severe acidosis induced contraction of both ED-intact and ED-denuded thoracic aorta rings. B, C, pH-response fitting curves of the EC_50_ for SHRs and Wistar rat aorta rings (6.51 vs 7.11, *P*<0.05). *P<0.01, compared with the contraction at pH 7.4. ^#^ *P*<0.05, compared with the contraction at pH 6.4.

Severe and extreme acidosis induced contraction of both endothelium-intact (ED-intact) and endothelium-denuded (ED-denuded) thoracic aorta rings (Figures 1, 2), which suggested that this contraction was independent of endothelium (the following experiments involved mainly ED-denuded aorta rings). For normotensive Wistar rats, contractions at various pH did not differ: pH 6.4 (18.48±2.36%), 5.4 (18.67±2.38%) and 4.4 (18.08±1.96%) (Figure 2B). However, for SHRs, contractions were higher at pH 5.4 (127.46±10.85%) or 4.4 (126.73±6.74%) than at pH 6.4 (103.29±7.51%), with no difference between contractions at pH 5.4 and 4.4 (Figure 2A). At every pH, acidosis-induced contractions were greater for SHRs than normotensive Wistar rats (Figure 2A). pH-response fitting curves showed that the EC_50_ pH was lower for SHRs than Wistar rats (6.51 vs 7.11, *P*<0.05; Figure 2B, C).

### Role of Extracellular Calcium Influx in Severe and Extreme Acidosis-induced Contraction of Thoracic Aortas

**Figure 3:**
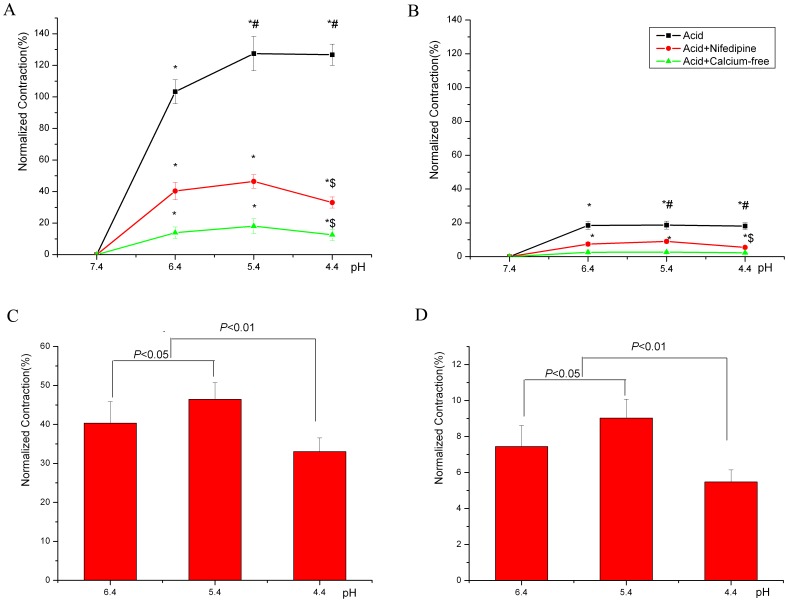
The role of extracellular calcium influx in severe acidosis-induced contraction of thoracic aortas from SHRs and Wistar rats. A, SHRs; B, Wistar rats: Effect of voltage-dependent calcium channel (VDCC) inhibitor nifedipine (10µM) on severe acidosis-induced contraction of thoracic aortas from SHRs (n = 6) and Wistar rats (n = 6) at different pHs and extracellular calcium-free solution. C, SHRs; D, Wistar rats: Effect of nifedipine on remnant contraction of thoracic aortas. *P<0.01, compared with the contraction at pH 7.4. ^#^ *P*<0.05, compared with the contraction at pH 6.4. ^$^ *P*<0.01, compared with the the contraction at pH 5.4.

Extracellular calcium-free solution inhibited acidosis-induced contraction of thoracic aortas at each pH (Figure 3A, B). The VDCC blocker nifedipine (10 µM) also inhibited severe acidosis-induced contraction in both SHRs and Wistar rats (Figure 3A, B). Pretreatment with extracellular calcium-free solution conferred no difference in remnant contractions at pH 6.4, 5.4 and 4.4 for both SHRs and Wistar rats (Figure 3A, B). However, with nifedipine, although the remnant contraction of aorta rings was higher at pH 5.4 than pH 6.4 for both SHRs and Wistar rats (Figure 3C, D), the remnant contraction was greatly reduced at pH 4.4 as compared with pH 6.4 and 5.4 (Figure 3C, D). At every pH, the remnant contractions were greater for SHRs than normotensive Wistar rats.

### Effect of I_Cl,acid_ Blockers on Severe Acidosis-induced Contraction of Thoracic Aortas

**Figure 4:**
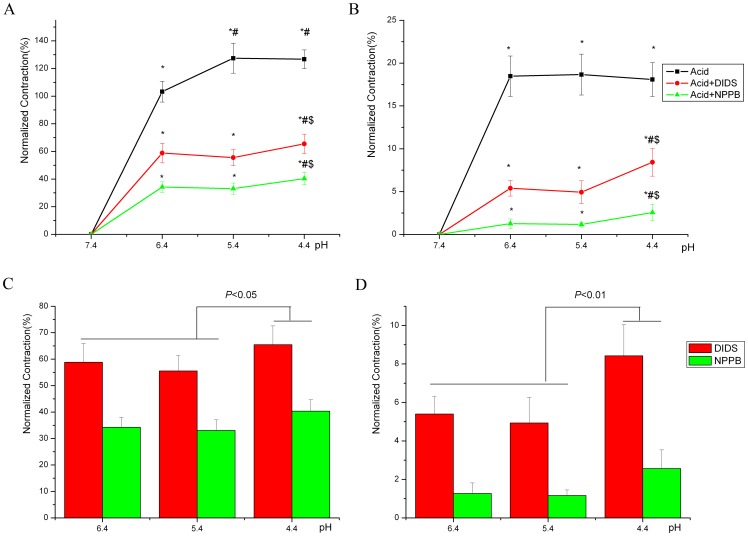
Effect of Cl^**−**^ channel inhibitors on severe acidosis-induced contraction of thoracic aortas from SHRs and Wistar rats. A, SHRs; B, Wistar rats: Effect of Cl^−^ channel blocker 4,4′-diisothiocyanatostilbene-2, 2′-disulfonic acid (DIDS; 100 µM) and 5-nitro-2-(3-phenylpropylamino) benzoic acid (NPPB; 100 µM) on severe acidosis-induced contraction of thoracic aortas from SHRs (n = 6) and Wistar rats (n = 6) at various pH levels. C, SHRs; D, Wistar rats: Effect of DIDS or NPPB on contractions at various pH levels. *P<0.01, compared with the contraction at pH 7.4. ^#^ *P*<0.05, compared with the contraction at pH 6.4. ^$^ *P*<0.05, compared with the contraction at pH 5.4.

The acidic pH**-**activated chloride channel blockers DIDS (100 µM) and NPPB (100 µM) inhibited severe acidosis-induced contraction of thoracic aortas from both SHRs and Wistar rats at different pH levels (Figure 4A, B), without affecting resting tensions under normal pH (data not shown). Cl^−^ channel blockers produced no difference between remnant contractions at pH 5.4 and 6.4; however, the remnant the contraction was greater at pH 4.4 than at pH 5.4 and 6.4 for both SHRs and Wistar rats (Figure 4C, D). The remnant contraction was still greater for SHR than normotensive Wistar aortas.

### Ratio of Remnant Contractions at pH 5.4 and 4.4

**Figure 5:**
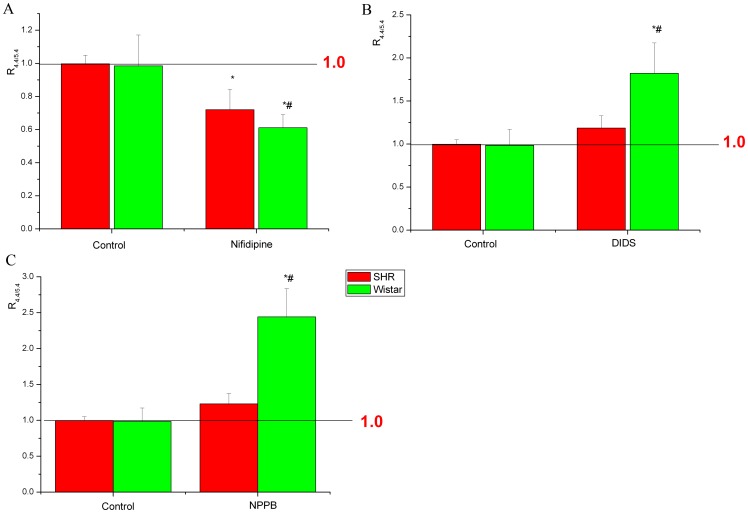
Remnant contraction of thoracic aortas for SHRs and Wistar rats with VDCC blocker nifedipine and I_Cl,acid_ blockers. Previous studies found that I_Cl,acid_ was usually activated at pH<5.5, so the ratio of the remnant contractions at pH 5.4 and 4.4 (R_4.4/5.4_) reflects whether the aorta rings contracted further with pH decreasing from 5.4 to 4.4. A, Effect of nifedipine on R_4.4/5.4_ in SHRs and Wistar rats. Effect of I_Cl,acid_ blockers (B) DIDS and (C) NPPB on R_4.4/5.4_ in SHRs and Wistar rats. *P<0.01, compared with the control in both SHRs and Wistar rats. ^#^ *P*<0.01, compared with SHRs.

Because previous studies found that I_Cl,acid_ was usually activated at pH <5.5, the ratio of the remnant contractions at pH 5.4 and 4.4 (R_4.4/5.4_) reflects whether the aorta rings contract further with pH decreasing from 5.4 to 4.4. With I_Cl,acid_ blockers, R_4.4/5.4_ was greater for both SHRs and Wistar rats than the control (Figure 5B,C), which suggested that the aorta contracted further with pH decreasing from 5.4 to 4.4. Furthermore, with I_Cl,acid_ blockers, R_4.4/5.4_ was lower for SHRs than Wistar rats. In contrast, with nifedipine, R_4.4/5.4_ was lower for both SHRs and Wistar rats than the control, and R_4.4/5.4_ was greater for SHRs than Wistar rats (Figure 5A).

### Effect of Severe and Extreme Acidosis on [Ca^2+^]_i_ and I_Cl,acid_ in Aortic SMCs

**Figure 6:**
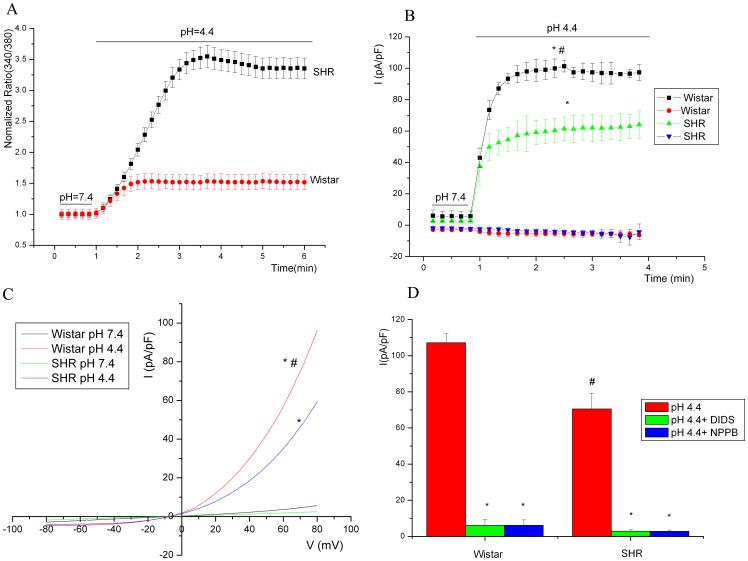
Severe acidosis increased intracellular calcium concentration and outward rectifying chloride channel currents in aorta smooth muscle cells (SMCs) from SHRs and Wistar rats. A, pH 4.4 solution increased intracellular calcium concentration in SMCs from SHRs as compared with Wistar rats. B, Mean current recordings at +80 and –80 mV membrane potentials obtained from voltage ramps during incubation with acidic solutions. C, Corresponding current–voltage relationships at the last sweep of voltage ramps showing the outward current with a clear outward rectification. D, The chloride channel blockers DIDS (100 µM) and NPPB (100 µM) abolished the outward current activated at pH 4.4. *P<0.01, compared with the control in both SHRs and Wistar rats. ^#^ *P*<0.01, compared with SHRs.

pH 4.4 solution-induced increase in [Ca^2+^]_i_ was greater in SMCs from SHRs than Wistar rats (Figure 6A). However, pH 4.4-induced currents were lower in SMCs from SHRs than Wistar rats (Figure 6B, C). Current–voltage relationships showed that the outward current exhibited a clear outward rectification (Figure 6C). The chloride channel blockers DIDS (100 µM) and NPPB (100 µM) completely abolished the outward current activated at pH 4.4 (Figure 6D). These characteristics were in agreement with I_Cl,acid_ findings described previously.

**Figure 7:**
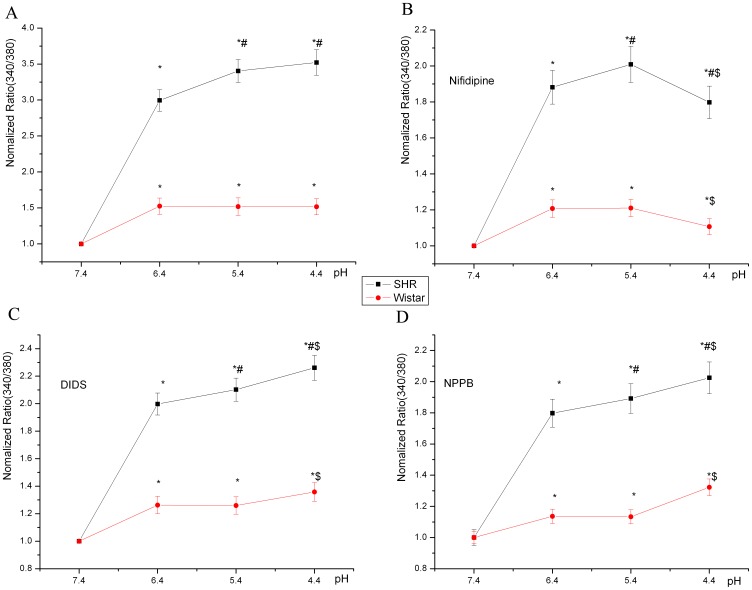
Effect of I_Cl,acid_ and VDCC blocker on severe acidosis-increased intracellular calcium concentration. A Without any treatment, [Ca^2+^]_i_ at pH 6.4, 5.4 and 4.4 in SMCs from SHRs and Wistar rats. B, VDCC blocker nifedipine (10 µM) inhibited severe acidosis-increased [Ca^2+^]_i_ for both SHRs and Wistar rats at different pHs. C, (DIDS, 100 µM), D, (NPPB, 100 µM), I_Cl,acid_ blockers inhibited severe acidosis-increased [Ca^2+^]_i_. *P<0.01, compared with [Ca^2+^]_i_ at pH 7.4. ^#^ *P*<0.05, compared with [Ca^2+^]_i_ at pH 6.4. ^$^ *P*<0.01, compared with [Ca^2+^]_i_ at pH 5.4.

Without any drugs, [Ca^2+^]_i_ did not differ at pH 6.4, 5.4 and 4.4 in SMCs from Wistar rats, but [Ca^2+^]_i_ was greater at pH 5.4 and 4.4 than at pH 6.4 in SMCs from SHRs (Figure 7A). Nifedipine (10 µM), DIDS (100 µM) and NPPB (100 µM) inhibited severe acidosis-increased [Ca^2+^]_i_ for both SHRs and Wistar rats at different pH levels (Figure 7B, C, D). With nifedipine, the remnant [Ca^2+^]_i_ was greatly reduced at pH 4.4 as compared with pH 6.4 and 5.4. In contrast, with I_Cl,acid_ blockers, [Ca^2+^]_i_ did not differ at pH 5.4 and 6.4 but was greater at pH 4.4 than at pH 5.4 and 6.4 for both SHRs and Wistar rats. Trypan blue exclusion demonstrated that solutions at each pH with or without drugs did not reduce the viability of SMCs from Wistar rats (Figure S1).

## Discussion

The homeostasis of extracellular pH (pH_o_) is important for maintaining cardiovascular function [2], [12]. Increasing evidence has revealed that extracellular acidosis could modulate vascular tone and play an important role in hypertension [3]–[5]. In the present study, severe and extreme acidosis induced contractions of both ED-intact and -denuded thoracic aorta rings from both SHRs and Wistar rats, which suggested that this contraction was independent of endothelium. pH 6.4 solution induced significant contraction of thoracic aortas from both SHRs and Wistar rats, which was similar to most previous findings [3], [5].

However, Celotto *et al.* [4] found that extracellular acidification (pH 6.5) had no effect on arteries from Wistar rats with or without endothelium pre-contracted with KCl (45 mM) and that extracellular acidosis caused pH-dependent relaxation in ED-intact and -denuded aorta rings pre-contracted with phenylephrine. Recently, local acidosis was found to likely contribute to functional sympatholysis by opposing sympathetically mediated vasoconstriction without affecting vasodilatation [13]. Acidosis was also found to attenuate P2X purinergic vasoconstriction in skeletal muscle arteries [14]. pH had no effect on phenylephrine dose–response curves [13], [14]. These results suggest that extracellular acidosis attenuates receptor-induced contraction rather than KCl-induced contraction and that acidosis has no effect on vasodilatation. Previous studies have shown that acidic pH induced greater contraction in aortas from SHRs than from normotensive rats [3], [5]. The findings all suggest that severe acidosis can induce contraction of aortas in hypertension and contribute to functional sympatholysis.

Celotto *et al.* did not investigate effect of pH 6.5 solution on the resting tension of Wistar rat aortas. Our study provided new findings that extreme and severe acidosis induced contraction of Wistar rat aortas. Most previous studies studied the effect of only severe acidosis (pH 6.5) on contractions of thoracic aortas from SHRs and normotensive rats. So we decreased the pH further to 5.4 or 4.4 and found that thoracic aortas from Wistar rats did not contract further under extreme acidosis. However, thoracic aortas from SHRs contracted more at pH 5.4 or 4.4 than at pH 6.4. The results suggest that aorta may be protected against excessive vasoconstriction in extreme acidosis in normotensive rats, and this protection may be reduced in hypertension.

The mechanism of acidosis-induced artery contraction is usually considered intracellular calcium elevation in SMCs by influx from extracellular solution or release from the sarcoplasmic reticulum [15], [16]. We found that the VDCC blocker nifedipine (10 µM) inhibited severe acidosis-induced contraction of thoracic aortas from both SHRs and Wistar rats. Moreover, in extracellular calcium-free solution, the acidosis-induced contraction was largely inhibited at each pH. We also found that severe acidic solution increased [Ca^2+^]_i_ in SMCs from both SHRs and Wistar rats, which could be inhibited by nifedipine. These results suggest that calcium influx through the VDCC plays a key role in severe acidosis-induced artery contraction [16].

However, we have no evidence that acidosis directly activates VDCC. The mechanisms involved in this response are not completely understood. Previously, the contraction induced by acidic pH (6.5) in the isolated aorta was found to be partially mediated by the activation of Cl^−^ channels [5]. More recently, a novel type of chloride channel activated by severe acidic solution was found in various mammalian cell types [6]–[8]. This channel was activated by very acidic extracellular conditions (pH <5.5) and was independent of intracellular Ca^2+^. Our previous study also found this channel in human endothelial cells [9]. However, whether this channel plays an important role in the reactions of rat thoracic aorta to severe acidosis is unclear. In the present study, we found this channel in isolated aortic SMCs. I_Cl,acid_ blockers (NPPB or DIDS) inhibited severe acidosis-induced contraction of aortas at different pH levels, without affecting the resting tensions for both SHRs and Wistar rats under normal pH. The mechanism might be that DIDS produced a relaxant effect on the acidosis-induced contraction by inhibiting background Cl^−^ channels, thus leading to hyperpolarization and the closing of VDCC in SMCs [17], [18]. We also revealed that I_Cl,acid_ blockers could inhibit pH 4.4 acidic solution-increased [Ca^2+^]_i_, which confirmed this mechanism.

Most interesting of our study was that the contraction was not increased with decreasing pH from 5.4 to 4.4 in Wistar rats. Some factor may hinder arteries from contracting further at pH 5.4 to 4.4. When blocking I_Cl,acid_, remnant contractions did not differ at pH 5.4 and 6.4; however, the remnant contraction was greater at pH 4.4 than at pH 5.4. Therefore, the thoracic aorta contracted further in normotensive Wistar rats without I_Cl,acid_. In contrast, with the VDCC blocker in Wistar rats, the remnant contractions were lower at pH 4.4 than at pH 5.4 and were even lower than at pH 6.4. Because I_Cl,acid_ is activated by very acidic extracellular pH (pH <5.5) [6]–[9], I_Cl,acid_ may protect the normal artery against excess vasoconstriction under extremely acidic conditions. This mechanism is important for maintaining normal vascular function under some pathological conditions such as ischemia [19], hypoxia [20], and metabolic disorders [21] causing local or systemic extracellular acidification.

To investigate whether this protective effect changed in hypertention, we defined a new measurement: the ratio between remnant contractions at pH 4.4 and 5.4 (R_4.4/5.4_), which reflected whether the aorta rings contracted further with pH decreasing from 5.4 to 4.4. With R_4.4/5.4_>1, the aorta rings contracted further from pH 5.4 to 4.4. Without any ion channel blocker, the mean R_4.4/5.4_ was about 1, so the aorta rings did not contract further. With nifedipine blockage, R_4.4/5.4_ was lower for both SHRs and Wistar rats than the control, and the R_4.4/5.4_ was lower for Wistar rats than SHRs in the presence of I_Cl,acid_. However, when blocking I_Cl,acid_, the R_4.4/5.4_ was higher for SHRs than Wistar rats. Furthermore, the mean current of I_Cl,acid_ in SMCs was lower for SHRs than Wistar rats. These results suggest that the protective effect of I_Cl,acid_ on the artery was decreased in hypertension.

Although it is not certain whether the pH of the internal environment can be lower than 5.5, previous studies have found that acidosis is usually accompanied by other internal environmental disorders such as hypoxia [22], oxidative stress [23], and changing temperature [24]. These factors might modify characteristics of ion channels [25], [26]. For example, the human muscle ClC-1 chloride channel depends on temperature [27]. Swelling-activated Cl current could be regulated by angiotensin II signaling and NADPH oxidase in the rabbit ventricle [28]. Moreover, the threshold pH to activate I_Cl,acid_ was found to increase with increasing environmental temperature [29]. So I_Cl,acid_ may be able to change its properties through some intra- or extracellular signaling events to become activated under slightly acidic environments. Further studies are needed to examine this hypothesis.

## Conclusions

We found that acidic pH-activated chloride channel (I_Cl,acid_) could protect rat arteries against excess vasoconstriction under extremely acidic extracellular conditions. This protective effect of I_Cl,acid_ was decreased in SHRs. Because hypertension is usually complicated by ischemia and metabolic disorders, resulting in local or systemic acidosis [30], the protective effect of I_Cl,acid_ could be improved to avoid acidosis-induced injury to target organs. However the molecular structure and regulation of I_Cl,acid_ is still unclear, and more studies are needed to reveal the molecular biological characteristics and pathophsiological mechanism of this channel in hypertension.

## Supporting Information

Figure S1 **Viability of smooth muscle cell from Wistar rats.** A, Extreme and severe acidosis had no effect on cell viability. B, C; Blockers did not reduce viability at each pH.
(TIF)

Methods S1 **Supporting methods.**
(DOC)
