# Supplementary material for: Protective Role of Acidic pH-Activated Chloride Channel in Severe Acidosis-Induced Contraction from the Aorta of Spontaneously Hypertensive Rats
Source: PLoS One. 2013 Apr 8;8(4):e61018. doi: 10.1371/journal.pone.0061018 (PMC3620281; doi:10.1371/journal.pone.0061018)
Supplement: Methods S1 — Supporting methods. (DOC) [file pone.0061018.s002.doc]

**Supporting Information Methods**

Evaluation of Cell Viability

Smooth muscle cells were isolated from thoracic aorta of Wistar rats. Cell viability was assessed by trypan blue exclusion assay. Cells were exposed to acidosis and different drugs according as described in Methods. At the end of exposure, 20 µL of 0.4% trypan blue (Sigma-Aldrich) was added to the cell suspension. After about 5 min of equilibration, cells were counted under a microscope. Dead cells were dyed clear blue, and living cells were colorless. Cell viability (percentage of living cells, %) = number of living cells/(total number of living cells + dead cells) × 100%.
